# Linking Socioeconomic Status With Adolescent Nutrition: Analyzing Dietary Patterns and Micronutrient Deficiencies Among Adolescents Aged 13-17 Years in Lahore

**DOI:** 10.7759/cureus.83622

**Published:** 2025-05-06

**Authors:** Mnahil Moazzam, Qaisar Raza, Mehran Khan, Zoha Sohail, Ayesha Tariq Sindhu, Rakhshanda Batool, Kinza Imran, Sania Khalid, Iftikhar Younis Mallhi, Shahana Bashar

**Affiliations:** 1 Nutrition Department, Allied Health Sciences, Akhtar Saeed Medical and Dental College, Lahore, PAK; 2 Department of Food Science and Human Nutrition, University of Veterinary and Animal Sciences, Lahore, PAK; 3 Orthopaedics, Services Hospital Lahore, Lahore, PAK; 4 Food and Nutrition Department, Institute of Allied Health Sciences, Pakistan Kidney and Liver Institute, Lahore, PAK; 5 Nutrition Department, Afro-Asian Institute, Lahore, PAK; 6 College of Applied Health Sciences, A’Sharqiya University, Ibra, OMN; 7 Department of Human Nutrition and Dietetics, Riphah International University, Lahore, PAK; 8 School of Human Nutrition and Dietetics, Minhaj University Lahore, Lahore, PAK

**Keywords:** dietary determinants, dietary patterns, eating habits, malnutrition, micronutrient deficiencies, nutritional status, socioeconomic status

## Abstract

Background and objective

Micronutrient deficiencies affect adolescents worldwide, particularly those from low socioeconomic backgrounds. These deficiencies can lead to significant health and developmental challenges. Understanding the relationship between dietary determinants and micronutrient deficiencies is critical for developing effective interventions. This study aimed to evaluate the relationship between micronutrient deficiency symptoms (related to calcium, iron, vitamin A, and zinc), nutritional status, and dietary determinants among adolescents of lower- and upper-middle-class socioeconomic backgrounds.

Methods

This cross-sectional study was conducted among 230 adolescents aged 13-17 years, with 115 participants each selected from two private schools catering to upper and lower middle socioeconomic populations in Lahore, Pakistan. Socioeconomic status (SES) was measured based on the monthly household income of the participants. Data were collected using a questionnaire. Statistical analysis was performed using SPSS Statistics version 25 (IBM Corp., Armonk, NY).

Results

Our study found that 65.5% of participants from the lower-middle socioeconomic background were underweight, compared to 22.6% in the upper-middle socioeconomic group. Micronutrient deficiency symptoms such as easy fatigue (65%), koilonychia (66%), brittle/fragile nails (61%), muscle cramps (53%), and PICA (eating non-food substances) (55%) were significantly more common in the lower-middle socioeconomic group (p<0.05). Lower SES individuals consumed fewer nutrient-dense foods, with 51.3% rarely eating whole grains, and more processed items like carbonated beverages (22.6%) and fried foods (31.3%).

Conclusions

The study reveals that adolescents from the lower-middle socioeconomic group are more likely to be underweight and stunted, with micronutrient deficiencies linked to reduced consumption of nutrient-rich foods. This highlights the significant role of socioeconomic factors in shaping dietary behaviors and nutritional health.

## Introduction

Malnutrition refers to a widespread issue that may manifest in many different forms, such as undernutrition, stunting, wasting, micronutrient deficiencies, moderate to severe underweight, or thinness, as well as overweight and obesity. Improving nutrition has the potential to have a significant and beneficial effect on many facets of development, including poverty, environmental sustainability, and peace and stability [1]. Micronutrient deficiencies are characterized by inadequacies in key minerals and vitamins that the body demands in limited quantities for optimal growth and well-being [2].

Micronutrient deficiencies have a major detrimental impact on adolescents' physical and mental development, susceptibility to disease, including osteoporosis, and general productivity. It is also known as "hidden hunger." According to the World Health Organization (WHO), micronutrient deficiency affects approximately two billion individuals globally [3]. Hidden hunger commonly leads to long-term health effects and mental development issues, particularly in low-income children and women. A growing fraction of the worldwide population is now on the verge of hidden hunger due to rising food prices and climate change, which might have an impact on global health and economic growth [4-6].

Due to insufficient micronutrient consumption, around 12.5% of the world's population is malnourished, with 852 million individuals residing in underdeveloped countries. Iron, iodine, vitamin A, and zinc deficiencies are most frequent in women of reproductive age and children. These deficiencies are the consequence of a complex relationship of dietary, social, and environmental factors [7]. The 2018 Pakistani National Nutrition Survey found that 40.2% of children under the age of five have stunted growth, 17.7% are wasting, and 54% have anemia. Furthermore, 52% are vitamin A-deficient, 63% are vitamin D-deficient, 28.6% have iron deficiency anemia, and 18.6% are zinc-deficient. Underweight adolescents account for 21.1% in males and 11.8% in females. Over half of the adolescent girls (56.6%) have anemia, with 0.9% having severe anemia [8].

Pakistan suffers from a significant malnutrition burden, encompassing both undernutrition and overnutrition. According to the Pakistan Demographic and Health Survey (2017-18), stunting affects 38.9% of children, while 18.9% are underweight and 7.1% are wasting. This data highlights the severity of the country's malnutrition crisis [9]. The United Nations' Sustainable Development Goals, specifically Goal 2, prioritize addressing malnutrition, including micronutrient deficiencies, to eradicate hunger, establish the availability of food, boost nutrition, and encourage global agricultural sustainability [10].

The consumption of four food groups among adolescents and young adults, focusing on socioeconomic and cultural characteristics, was examined in a study related to the Belgian Food Consumption Survey. The research involved two 24-hour dietary recalls of 1,505 participants aged 10-39 years. After adjustment, it was found that adolescents in less educated households consumed lower amounts of fruits and vegetables and whole grain bread and cereals, and higher amounts of sugary sweetened beverages (SSB) compared to those in more educated households. The same trends were observed in older groups, with consumption disparities also observed by region of residency, country of birth, and occupation [11].

Adolescents are in a crucial stage of growth and development, making it important to identify signs of micronutrient deficiencies, especially for those from lower socioeconomic backgrounds who might struggle to get the right nutrition. In Pakistan, dietary practices are heavily influenced by socioeconomic constraints, cultural food taboos, and unequal access to diverse and nutrient-rich foods. Adolescents from lower socioeconomic backgrounds often rely on energy-dense, low-nutrient diets due to affordability and availability. Additionally, limited awareness of balanced nutrition, lack of nutrition education in school curricula, and gendered norms around food distribution within households further exacerbate deficiencies. Structural challenges such as inflation, food insecurity, and fragmented school health programs also hinder sustainable improvements in adolescent nutritional status.

The purpose of the current study is to determine the prevalence of micronutrient deficiency symptoms (related to calcium, iron, vitamin A, and zinc) among adolescents from low and high socioeconomic backgrounds. We also aim to evaluate the relationship between micronutrient deficiency symptoms, nutrition status, and dietary determinants in adolescents from low and high-socioeconomic status (SES) groups.

## Materials and methods

Study design

This was a cross-sectional study design conducted through a structured questionnaire.

Sample size

The formula used for sample size calculation is clearly stated in the manuscript: n = (Z^2 * p * (1-p)) / d^2

The formula used in the sample calculation factored in a confidence level (z) of 95%, a margin of error (d) of 6%, and an expected percentage of 31.8%. The resulting minimum sample size was 230 adolescents [12]. Eventually, 115 students were selected from schools of low SES and 115 from high SES.

Sampling technique

In this study, the convenience sampling technique was employed, which entailed selecting participants who were readily accessible and willing to participate, which in this case included students present at selected schools during data collection. As it was a cross-sectional study, data were collected by using a structured questionnaire. Micronutrient deficiency was identified by Nutrition-focused physical examination (NFPE). The nutritional status of adolescents was assessed by anthropometric measurements, and dietary determinants were investigated by food frequency questionnaire (FFQ) and 24-hour recall.

Study setting

Data were collected from different private schools of Lahore, catering to lower- and upper-middle classes (Table 1). 

**Table 1:** List of schools

Sr. no.	Lower middle class	Upper middle class
1.	Punjab Central School	American Lycetuff
2.	Tameer-e-Nau	Allied School

Inclusion and exclusion criteria

 The inclusion criteria were as follows: adolescents aged 13-17 years.

 The exclusion criteria were as follows:

• Individuals under the age of 13 years and over the age of 17 years.

• Bedridden individuals or those unable to be measured.

• Adolescents with critical illnesses and those whose parents refused to participate

Recruitment

Adolescents aged 13-17 years from low- and high-socioeconomic areas of Lahore were chosen voluntarily. According to World Bank assessment, the lower middle income group typically includes households with a monthly income ranging from 44,000PKR to 88,000PKR, while the upper middle income group includes those with monthly incomes from 88,00PKR to 170,000PKR. There were 115 adolescents recruited from each socioeconomic class.

All monetary values mentioned have been converted to and expressed in US dollars (USD).

44,000PKR = 157$

88,000PKR = 314$

170,000PKR = 607$

Ethical approval

Ethical approval was obtained from the Institutional Review Committee for Biomedical Research at the University of Veterinary and Animal Sciences (reference number: 252/IRC/BMR).

Questionnaire development

The data were collected by using a structured and pre-validated questionnaire from adolescents. This questionnaire had six parts (sociodemographic, anthropometric measurements, nutrition-focused physical assessment, dietary behaviors, food frequency table, and 24-hour recall). We used a closed-ended questionnaire that was primarily focused on assessing micronutrient deficiencies, nutritional status, and dietary patterns of adolescents living in low- and high-socioeconomic areas.

In the sociodemographic section, information was collected about the adolescent's gender and age, the educational level of parents and participants, the occupation of parents, the total number of family members, and wealth status.

This questionnaire, particularly the anthropometric measurements section, collected key measurements such as weight, height, and, in addition, weight and height for age. It evaluated the participant's nutritional status, specifically weight for age and the presence of stunted growth (low height for age). CDC Growth charts were used to assess these parameters [13]. The growth charts' percentile ranges are presented in Table 2.

**Table 2:** Growth charts' percentile ranges

Sr. no.	Percentile ranges	Weight
1.	Less than 5 percentile	Underweight
2.	5-85 percentile	Normal weight
3.	85-95 percentile	Overweight
4.	Above 95 percentile	Obesity

The NFPE part is commonly recommended for registered dietitians to screen and identify physical signs and symptoms in adolescents and may indicate underlying micronutrient deficiencies. This includes evaluating a variety of criteria such as poor appetite, fatigue or weakness, skin condition, nail health, hair health, muscle and joint health, PICA (eating non-food substances), and any recurring infections or diseases. Furthermore, the appearance of specific indicators such as Bitot's spots and night blindness can provide crucial information concerning vitamin A deficiency and other micronutrient imbalances [14-16].

The "dietary behaviors" segment provides insights into the adolescents' daily food consumption patterns. This component assesses the dietary habits of the adolescent by examining how many meals they generally consume in a day and whether meal skipping is widespread.

The "food frequency table" plays an important role in gathering concise and quantitative data on the dietary patterns of adolescents from low- and high-SES households in Lahore, Pakistan. This section examined the frequency with which key food groups, such as whole grains, legumes, meats, dairy products, nuts, vegetables, and fruits, are consumed [17-18].

The "24-hour recall" portion was of great assistance because it provides a detailed analysis of the adolescent's recent dietary practices, including breakfast, lunch, dinner, and any snacks. This extensive dietary-related data provide the total calorie intake, and identify potential dietary gaps or excesses. It also helped in the identification of any specific dietary imbalances that may necessitate quick correction.

Pilot study

Before data collection, the questionnaire was pilot-tested with a group of 30 adolescents from both socioeconomic backgrounds to assess its clarity, feasibility, and relevance for the study population. The pilot study aimed to assess the clarity and appropriateness of the questionnaire, particularly the NFPE, and refine it based on feedback to ensure accurate data collection in the main study. The pilot helped validate the reliability of our questionnaire, particularly for clarity, cultural relevance, and feasibility. The Cronbach’s alpha was calculated for internal consistency and yielded a satisfactory value of 0.81.

Statistical analysis

Data were tabulated and analyzed with the help of statistical software SPSS Statistics version 25 (IBM Corp., Armonk, NY). Descriptive statistics were used to present frequencies and percentages of micronutrient deficiencies, nutritional status, and dietary habits. Pearson’s chi-squared test was applied to determine the relationship between dietary determinants, anthropometric measurements, and micronutrient deficiencies.

## Results

Sociodemographic data

In the lower-middle SES group, there were 58 (50.4%) male participants and 57 (49.7%) female participants. However, in the upper-middle SES group, there were 60 (52%) male participants and 55 (47%) female participants. Table 3 compares the demographic data of participants from lower- and upper-middle SES groups, each consisting of 115 participants. 

**Table 3:** Comparison of sociodemographic data SES: socioeconomic status

Variables	Lower-middle SES group (n=115)		Upper-middle SES group (n=115)	
	Frequency (N)	Percentage (%)	Frequency (N)	Percentage (%)
Family members				
1-4	21	18.3	81	70.4
5-8	72	62.6	28	24.3
Above 8	22	19.1	6	5.2
Father’s education				
Illiterate	38	33	6	5.2
Primary school	26	22.6	3	2.6
Matriculation	19	16.5	7	6.1
Secondary level	17	14.8	16	13.9
Bachelor's	8	7	39	33.9
Above bachelor's	7	6.1	44	38.3
Mother’s education				
Illiterate	19	16.5	5	4.3
Primary school	22	19.1	8	7.0
Matriculation	36	31.3	9	7.8
Secondary level	22	19.1	33	28.7
Bachelor's	9	7.8	44	38.3
Above bachelor's	7	6.1	16	13.9
Father’s occupation				
Self employed	28	24.3	52	45.2
Govt. employee	9	7.8	41	35.7
Pvt. employee	65	56.5	22	19.1
Unemployed	13	11.3	0	0.0
Mother’s occupation				
Housewife	83	72.2	57	49.6
Self employed	11	9.6	16	13.9
Govt. employee	4	3.5	13	11.3
Pvt. employee	17	14.8	29	25.2

Anthropometric measurements

**Figure 1:**
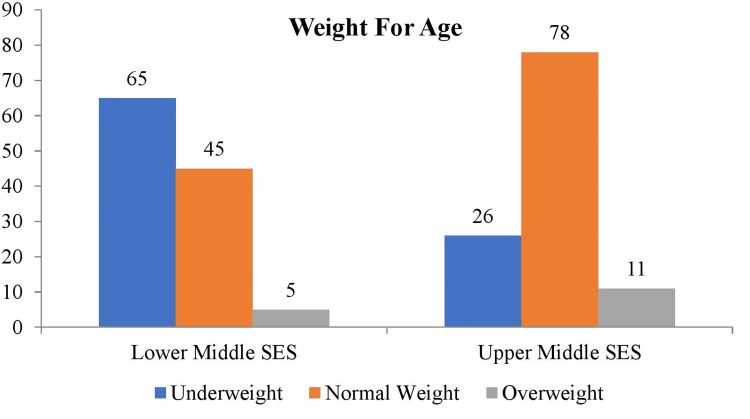
Frequency distribution of weight for age among participants Descriptive stats applied and values are represented in frequency (N) SES: socioeconomic status

As highlighted in Figure 1, there was a higher prevalence of underweight individuals [n=65 (56%)] in the low SES group and a higher prevalence of normal weight individuals [n=78 (67.8%)] in the high SES group.

**Figure 2:**
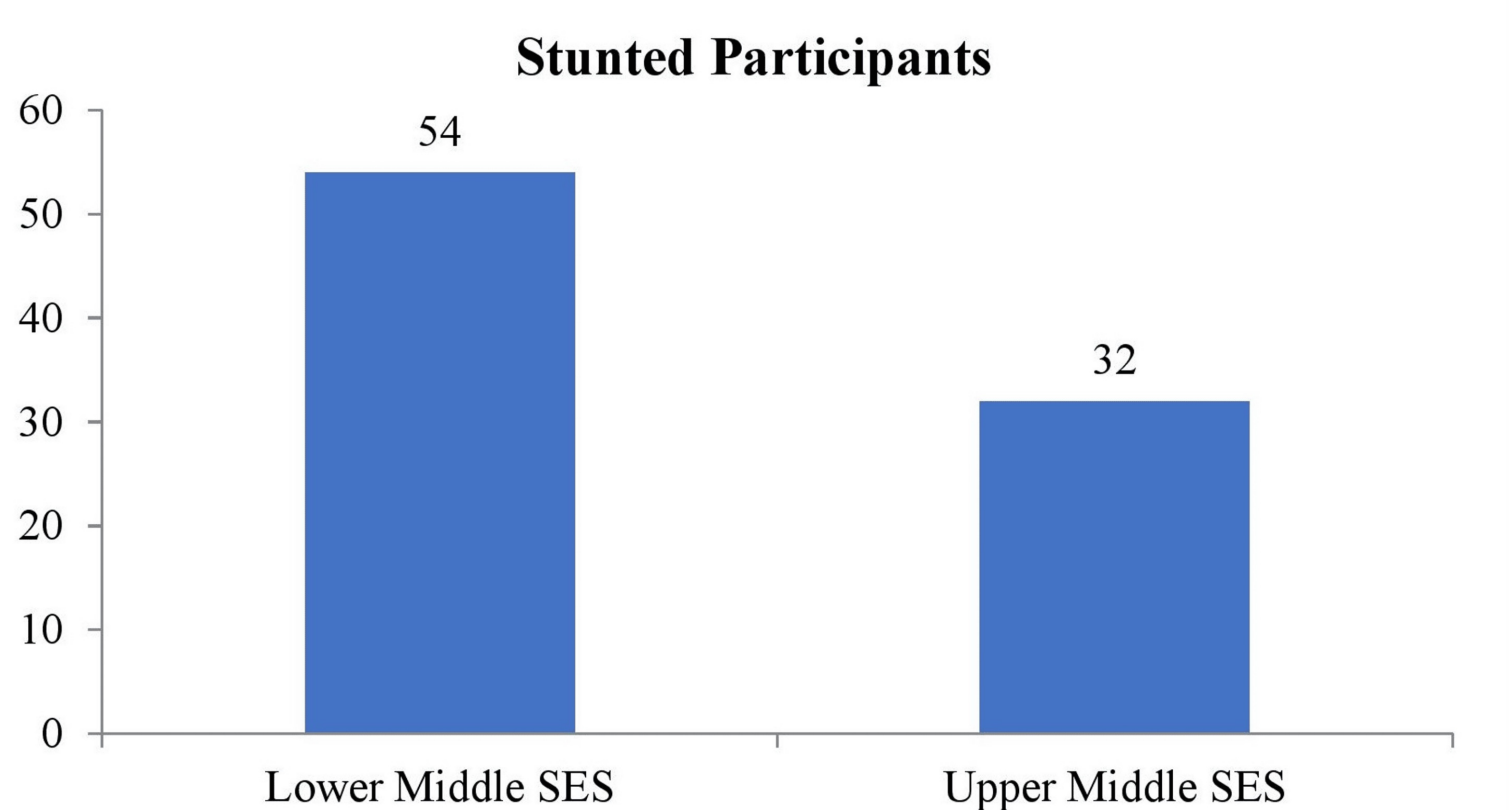
Frequency distribution of height for age among participants Descriptive stats applied and values are represented in frequency (N) SES: socioeconomic status

Figure 2 shows the prevalence of stunting among participants of different SES. In the low socioeconomic group, 54 (46.9%) adolescent students were stunted, compared to 32 (27.8%) in the high socioeconomic group.

Nutrition-focused physical assessment

**Figure 3:**
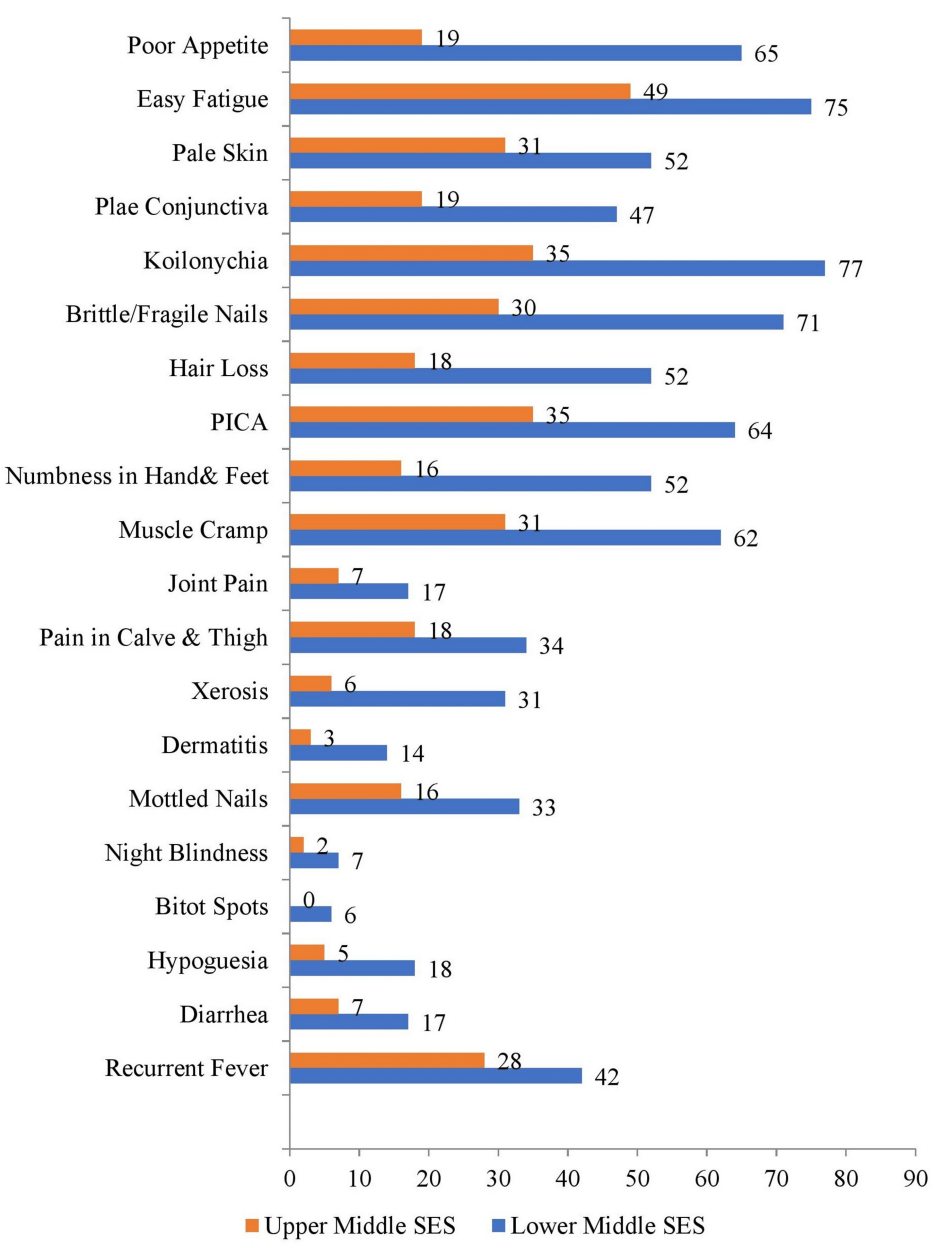
Nutrition-focused physical examination Descriptive stats applied and values are represented in frequency (N) SES: socioeconomic status

Figure 3 and Table 4 present a comparison of micronutrient deficiency symptoms between lower- and upper-middle SES groups. In the low SES group, 65 (56.5%) participants reported poor appetite, 75 (65%) experienced easy fatigue, and 52 (45%) had pale skin. Koilonychias affected 77 (67%) in the lower middle SES group, and 64 (55%) showed PICA symptoms. In the upper middle SES group, 19 (16.5%) participants reported poor appetite, 75 (42.6%) experienced easy fatigue, and 52 (27%) had pale skin. Brittle nails were seen in 71 (61.7%) in the low SES group versus 30 (26%) of the upper-middle SES group.

**Table 4:** Association between micronutrient deficiency symptoms and socioeconomic status The chi-squared test was applied to compare the micronutrient deficiency symptoms between socioeconomic groups. P-values indicate statistically significant differences (p<0.05) df: degree of freedom; χ²: Pearson chi-square value

Micronutrient deficiency symptoms	χ²	df	P-value
Poor appetite	40	1	0.00
Easy fatigue	12	1	0.00
Pale skin	8.4	1	0.00
Pale conjunctiva	17	1	0.00
Koilonychias	31	1	0.00
Brittle/fragile nails	30	1	0.00
Hair loss	24	1	0.00
PICA	15	1	0.00
Numbness in hand and feet	27	1	0.00
Muscle cramp	17	1	0.00
Joint pain	4.6	1	0.03
Pain in calves and thighs	7	1	0.01
Xerosis	20	1	0.00
Dermatitis	7.7	1	0.00
Mottled nails	7	1	0.00
Night blindness	3	1	0.08
Bitot's spots	6	1	0.01
Hypoguesia	8	1	0.00
Diarrhea	4.6	1	0.00
Recurrent fever	4	1	0.04

Other symptoms, such as numbness [n=52 (45.2%) vs. n=16 (13.9%)], muscular cramps [n=62 (53.9%) vs. n=31 (27%)], and joint pain [n=7 (14.8%) vs. n=17 (6.1%)], were also more common in the lower-middle SES group. Xerosis was reported by 31 (33%) in the lower-middle SES group compared to six (5.2%) in the upper-middle SES group, and night blindness affected seven (8.7%) in the lower-middle SES group versus two (1.7%) in the upper-middle SES group.

Dietary behaviors

**Figure 4:**
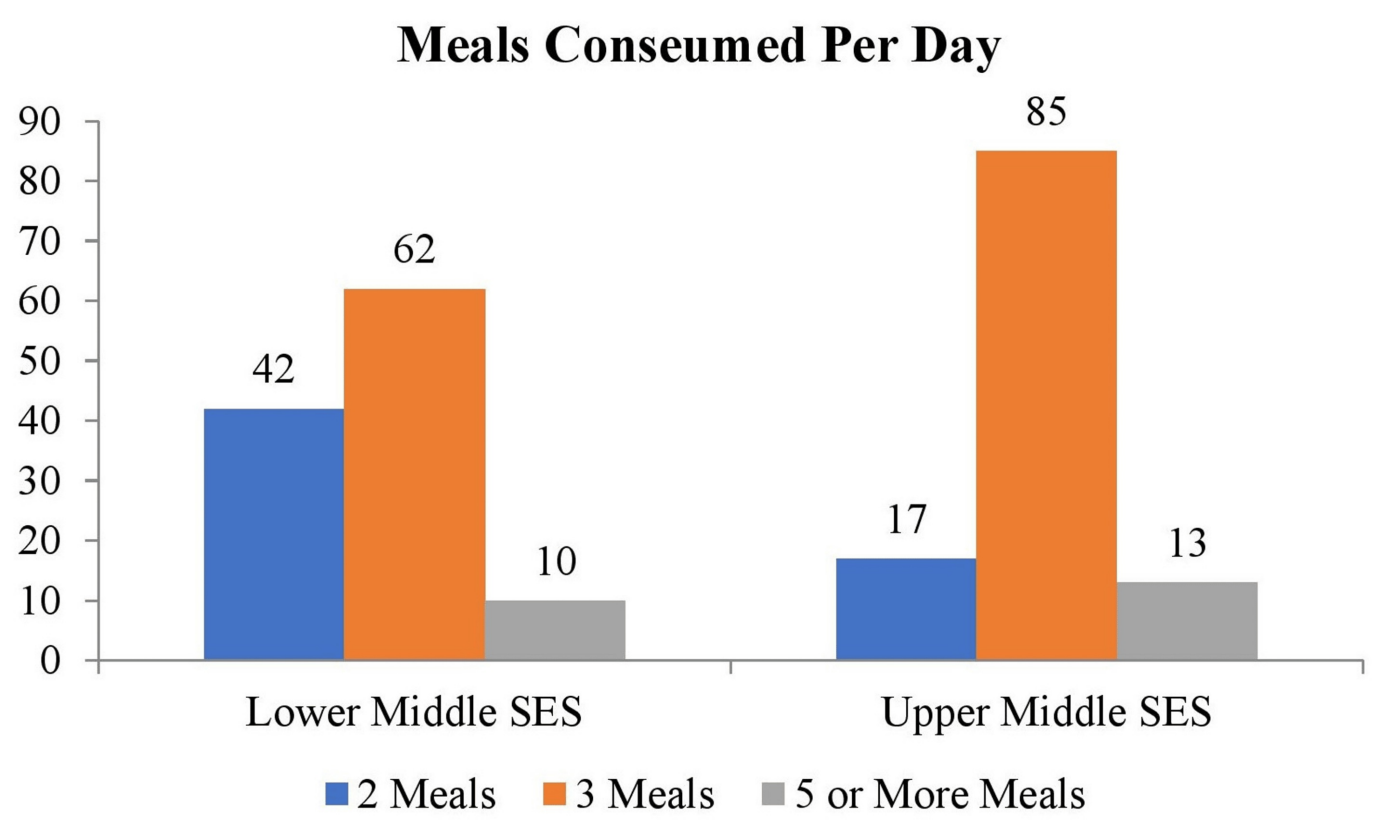
Frequency distribution of meals consumed per day Descriptive stats has been applied and values are represented in frequency (N) SES: socioeconomic status

Figure 4 shows the meal frequency distribution among lower- and upper-middle SES groups. In the lower SES group, 42 (36.5%) individuals consumed two meals per day, 63 (54%) consumed three meals, and 10 (8.7%) consumed five or more meals. In the upper-middle SES group, 17 (14.8%) consumed two meals, 85 (73.9%) consumed three meals, and 13 (11.3%) consumed five or more meals. The data provided significant results (p>0.05) with χ² 14.9 and degree of freedom of 2.

**Figure 5:**
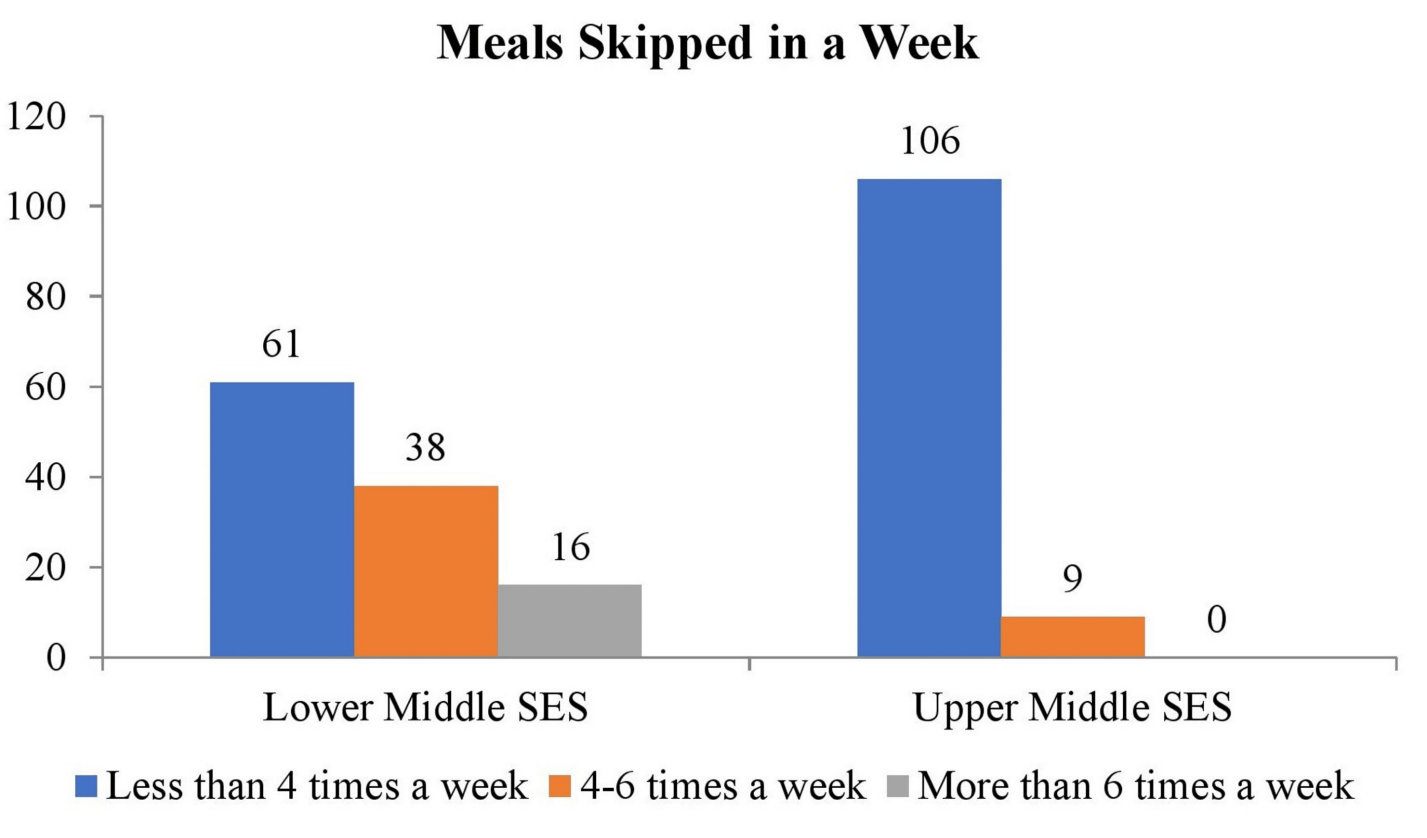
Frequency distribution of meals skipped in a week SES: socioeconomic status

Figure 5 shows the data on meal skipping frequency per week among the same SES groups. In the lower SES group, 61 (53%) participants skipped meals less than four times a week, 38 (33%) skipped four to six times, and 16 (14%) skipped more than six times. In the upper-middle SES group, 106 (92.2%) skipped meals less than four times a week, and nine (7.8%) skipped four to six times. These differences were also statistically significant (p<0.05) with χ² 46 and degree of freedom of 2.

Food frequency questionnaire

Table 5 highlights associations (p<0.05) between dietary intake and SES. There were significant dietary differences between lower-middle SES and upper-middle SES participants. Those from lower-middle SES consumed fewer whole grains, legumes, meat, dairy products, nuts, green leafy vegetables, and fruits compared to their upper-middle SES counterparts. In contrast, lower-middle SES individuals consumed more carbonated beverages, canned juice, sweets, and desserts, but less fast food and bakery items. Fried food consumption was higher in the lower-middle SES group.

**Table 5:** Association between dietary intake and socioeconomic status The chi-squared test was applied to compare the food consumption patterns between socioeconomic groups. P-values indicate statistically significant differences (p<0.05) High SES: upper-middle socioeconomic status; Low SES: lower-middle socioeconomic status

Variables	Never, n (%)	1-2 days/week, n (%)	3-4 days/week, n (%)	5-6 days/week, n (%)	Daily, n (%)		χ²	df		P-value
Whole grains										
Low SES	59 (51.3)	40 (34.8)	7 (6.1)	0 (0.0)	9 (7.8)	144			4	0.00
High SES	5 (4.3)	8 (7)	4 (3.5)	35 (30.4)	63 (54)					
Legumes										
Low SES	5 (4.3)	41 (35.7)	53 (46.1)	12 (10.4)	4 (3.5)		12.5	4		0.00
High SES	19 (16.5)	56 (48.7)	29 (25.2)	8 (7)	3 (2.6)					
Meat										
Low SES	12 (10.4)	88 (76.5)	10 (8.7)	5 (4.3)	0 (0.0)		168	4		0.00
High SES	8 (7)	4 (3.5)	38 (33)	45 (39.1)	20 (17.4)					
Dairy products										
Low SES	53 (46.1)	32 (26)	11 (8)	4 (3.4)	20 (13)		160	4		0.00
High SES	12 (10.4)	13 (11.3)	32 (27)	22 (19.1)	36 (31.3)					
Nuts										
Low SES	42 (36.5)	41 (35.7)	21 (18.3)	6 (5.2)	5 (4.3)		171	4		0.00
High SES	6 (5.2)	13 (11.3)	12 (10.4)	35 (30.4)	49 (42.6)					
Non-leafy vegetables										
Low SES	8 (7)	10 (8,7)	39 (33.9)	26 (22.6)	32 (27.8)		62.5	4		0.00
High SES	0 (0.0)	42 (36.5)	6 (5.2)	48 (41.7)	19 (16.5)					
Green leafy vegetables										
Low SES	30 (26.1)	57 (49.6)	17 (14.8)	8 (7.0)	3 (2.6)		182	4		0.00
High SES	0 (0.0)	14 (12.2)	36 (31.3)	45 (39.1)	20 (17.4)					
Fruits										
Low SES	18 (15.7)	67 (58.3)	11 (9.6)	11 (9.6)	8 (7.0)		188	4		0.00
High SES	4 (3.5)	3 (2.6)	21 (18.3)	22 (19.1)	65 (56.5)					
Carbonated beverages										
Low SES	26 (22.6)	21 (18.3)	11 (9.6)	43 (37.4)	14 (12.2)		12.2	4		0.01
High SES	46 (40.4)	23 (20.2)	11 (9.6)	29 (25.4)	5 (4.4)					
Canned juices										
Low SES	2 (1.7)	43 (37.4)	40 (34.8)	14 (12.2)	16 (13.9)		83.7	4		0.00
High SES	19 (16.5)	62 (53.9)	8 (7.0)	8 (7.0)	18 (15.7)					
Sweets/desserts										
Low SES	6 (5.2)	14 (12.2)	24 (20.9)	50 (43.5)	21 (18.3)		103	4		0.00
High SES	12 (10.4)	59 (51.3)	9 (7.8)	15 (13)	20 (17.4)					
Fast food										
Low SES	44 (38.3)	20 (17.4)	19 (16.5)	24 (20.9)	8 (7)		131	4		0.00
High SES	4 (3.5)	9 (7.8)	36 (31.3)	53 (46.1)	13 (11.30)					
Bakery items										
Low SES	37 (32.2)	60 (52.2)	6 (5.2)	12 (10.4)	0 (0.0)		74	4		0.00
High SES	24 (20.9)	14 (12.2)	16 (13.9)	33 (18.7)	28 (24.3)					
Fried foods										
Low SES	0 (0.0)	20 (17.4)	41 (35.7)	36 (31.3)	18 (15.7)		58	4		0.00
High SES	47 (40.9)	15 (13)	24 (20.9)	18 (15.7)	11 (9.6)					

## Discussion

The study aimed to determine the prevalence of micronutrient deficiency symptoms among adolescents from lower- and upper-middle SES. The relationship between nutrition status, micronutrient deficiency symptoms, dietary patterns, and SES has also been examined. Malnutrition is a major issue in Pakistan due to compromised dietary habits, specifically in low socioeconomic groups. Micronutrient deficiencies are more prevalent among adolescents belonging to low socioeconomic areas. In low-resource countries like Pakistan, child malnutrition is a serious problem, which emphasizes the necessity of policies and initiatives to address this vital public health concern. In Pakistan, more than 23% of children were underweight, 8% experienced wasting, and 37.7% had stunting, according to research looking at characteristics related to low weight, wasting, and stunting. Stunted, underweight, and wasted children were less common in high- and middle-class households.. The results point to the necessity of community-based education programs and focused public health initiatives to combat malnutrition in Pakistan [19].

The present study demonstrates the higher prevalence of underweight and stunted adolescent students in low socioeconomic areas. In low socioeconomic areas, 56.5% of participants are underweight, and 46.9% are stunted. Both stunting and low weight for age percentages are associated with poor dietary habits and compromised lifestyle due to insufficient resources. The compliance of adolescents to recommended dietary guidelines was determined in a study in Lahore, Pakistan. The research involved students from public and private high schools. The results showed that 46.2% of students were underweight, 2.3% were obese, and 6.2% were overweight. The study found that family and advertisements had the highest impact on adolescents' food choices, with scores of 6.5 ±2.69 and 6.1 ±2.77, respectively [20].

Along with SES, parental education level, occupation, and family members also play an important role in the growth and development of adolescents. In the current study, the literacy rate of parents was higher in high SES groups, which exerts a strong impact on the better growth and dietary habits of adolescents. However, in low SES groups, 33% of fathers and 16% of mothers were illiterate, which leads to a lack of nutrition education awareness and micronutrient deficiencies in adolescents. The symptoms of iron deficiency anemia (IDA) were evaluated by a study in Bahawalpur, Pakistan, among 500 university and college students. The results showed that 41.2% of the students were anemic. The majority of students were aged below 25 years, stayed in official hostels, and hailed from average socioeconomic backgrounds. Symptoms included flattened nails, dizziness, fatigue, glossitis, ringing in the ears, headaches, frequent minor infections, shortness of breath, taste disturbance, ice cravings, and angular stomatitis. [21]. About 56.5% of individuals in the low-middle SES group and 16% in the upper-middle SES group had a poor appetite. Around 65.2% of adolescents in the lower-middle SES group and 42.6% in the upper-middle SES group reported easy fatigue. In the lower middle SES group, there were about 45.2% of persons with pale skin.

A study investigating the prevalence of calcium deficiency symptoms in adolescent girls in Tamil Nadu, India, revealed that 72.7% of participants were not following proper nutritional diets and physical activities. The study also found that 63.6% had a calcium symptom score of 10. Common calcium deficiency symptoms include muscle cramps, muscle spasms, weak bones, and muscle pain. Calcium is crucial for bone, teeth, and soft tissues, and low intake during adolescence may increase the risk of osteoporosis [22]. The present study also assessed the symptoms of calcium deficiency among adolescents of low and high SES. Numbness and tingling in the hands and feet remained common, with 45.2% of the low SES group and 13.9% of the high SES group experiencing these symptoms. Muscular cramps were reported by 53.9% in the low SES group and 27.0% in the high SES group. Joint pain was noted in 14.8% of the low SES group and 6.1% of the high SES group. Additionally, discomfort in the thighs and calves affected 29.6% of the high SES group and 15.7% of the low SES group.

The prevalence of stunting, wasting, and thinness in children and adolescents varies across different regions. Factors such as low socioeconomic status, parental unemployment, lower maternal education, and rural dwelling are associated with higher stunting prevalence. Other predictors include larger family size, lower maternal education, poor dietary diversity, poor water, sanitation, and hygiene practices, and a history of illness. Vitamin A and vitamin D deficiency prevalence vary across countries, with higher rates in urban adolescents. Zinc deficiency prevalence is also higher, with a positive association between food insecurity and poverty. Dietary intakes and patterns vary across regions, with breakfast being skipped more often by girls and those living in rural areas [23].

According to this study, xerosis was reported by 33.0% in the low SES group and 5.2% in the high SES group, while dermatitis affected 12.2% in the low SES group and 2.6% in the high SES group. Furthermore, mottled nails were seen in 28.7% of the low SES group and 13.9% of the high SES group. Bitot spots were present in the eyes of 7.8% of the low SES group. Night blindness, indicating Vitamin A deficiency, affected 8.7% of the low SES group and 1.7% of the high SES group. Diarrhea, potentially signifying zinc deficiency, was reported by 14.8% of the low SES group and 6.1% of the high SES group. Periodic episodes of fever were experienced by 19.1% of the low SES group and 8.7% of the high SES group. Similarly, compromised immunity and susceptibility to infections were noted in 36.5% of the low SES group and 24.3% of the high SES group.

One study examined the relationship between whole grain consumption and anthropometric measures in children and adolescents. National Health and Nutrition Examination Survey data from 1999-2004 were used. Results showed that mean whole grain intake was 0.59 and 0.63 servings/day in children 6-12 years and 13.18 years, respectively. However, in adolescents, the highest whole grain consumption group had significantly lower BMI but not waist circumference [24]. This study found that food consumption patterns vary greatly between low and high SES groups. Individuals from low SES backgrounds are less likely to consume whole grains and nuts regularly but have higher meat and dairy consumption compared to those from upper-middle SES backgrounds. For example, 51.3% of lower-middle SES individuals never eat whole grains, while 54% of upper-middle SES individuals consume them daily. Legume consumption is also less frequent among lower-middle SES individuals, with 10.4% never eating them, compared to 2.6% in upper-middle SES.

Poor eating habits are widespread and frequently co-occur in 52 low- and middle-income countries (LMICs), according to a study involving 145,021 adolescents aged 12 to 15 years from these countries. The study assessed the prevalence of unhealthy dietary practices, such as consuming fast food, carbonated soft drinks, and consuming insufficient amounts of fruits and vegetables, using data from the Global School-Based Student Health Surveys conducted between 2009 and 2017. There were significant differences in the prevalence of these variables amongst the nations; the pooled prevalence of exposure to two or three suboptimal dietary factors was 20.0% and 41.8%, respectively [25].

The findings of this study showed that high SES individuals tend to consume carbonated beverages more frequently than those from lower-middle SES backgrounds, with 40.4% of upper-middle SES individuals drinking them daily compared to 22.6% from lower-middle SES. In contrast, canned juice consumption is more common among low SES individuals when compared to high SES individuals. Low SES individuals also consume sweets and desserts more often, whereas only 10.4% of high SES individuals do so. Additionally, fast food consumption is higher among low SES individuals but significantly higher than among high SES individuals.

Recommendations

To address nutritional disparities, authorities should implement comprehensive nutritional education to raise awareness about nutrient-rich foods, enhance school meal programs for balanced nutrition, and develop community-based support, including food subsidies and nutritional counseling for low SES families. Policy interventions should focus on reducing fast food and sugary drink consumption to promote healthier eating habits.

Limitations

This study has several limitations that must be acknowledged. Firstly, the sample size, while adequate for a cross-sectional design, limits the generalizability of findings to the broader adolescent population of Pakistan. Secondly, data were collected from schools within a single city (Lahore), which may not fully represent rural or regional diversity in dietary habits and socioeconomic dynamics. Thirdly, self-reported dietary data from 24-hour recall and FFQ may be subject to recall bias or under-/over-reporting, especially among younger participants.

Despite these limitations, the study offers valuable insights into the link between socioeconomic disparities and micronutrient deficiency risk among adolescents. However, the cross-sectional design of the study only provides a snapshot of data, necessitating longitudinal research to understand how SES, diet, and micronutrient deficiencies interact over time. Additionally, while a nutrition-focused physical assessment tool was used, biochemical testing would offer more precise and accurate identification of micronutrient deficiencies and their severity.

## Conclusions

This study demonstrates that adolescents from lower-middle socioeconomic backgrounds exhibited higher rates of underweight and stunting, alongside more frequent symptoms of micronutrient deficiencies, particularly in iron, calcium, vitamin A, and zinc. These deficiencies were linked to less frequent consumption of nutrient-rich foods such as whole grains, legumes, dairy products, and fruits. Conversely, adolescents from upper-middle socioeconomic backgrounds had better overall nutritional status and dietary patterns but still faced certain nutritional challenges, particularly in consuming fast foods and carbonated beverages. This study underscores the important role of socioeconomic factors in shaping dietary behaviors and nutritional health among adolescents.
